# Thai SF-36 health survey: tests of data quality, scaling assumptions, reliability and validity in healthy men and women

**DOI:** 10.1186/1477-7525-6-52

**Published:** 2008-07-18

**Authors:** Lynette L-Y Lim, Sam-ang Seubsman, Adrian Sleigh

**Affiliations:** 1 National Centre for Epidemiology and Public Health, Mills Road, Australian National University, Acton, ACT, 0200, Australia; 2 School of Human Ecology, Sukhothai Thammathirat Open University, Pakkret, Nonthaburi, 11120, Thailand

## Abstract

**Background:**

Since its translation to Thai in 2000, the SF-36 Health Survey has been used extensively in many different clinical settings in Thailand. Its popularity has increased despite the absence of published evidence that the translated instrument satisfies scoring assumptions, the psychometric properties required for valid interpretation of the SF-36 summated ratings scales. The purpose of this paper was to examine these properties and to report on the reliability and validity of the Thai SF-36 in a non-clinical general population.

**Methods:**

1345 distance-education university students who live in all areas of Thailand completed a questionnaire comprising the Thai SF-36 (Version 1). Median age was 31 years. Psychometric tests recommended by the International Quality of Life Assessment Project were used.

**Results:**

Data quality was satisfactory: questionnaire completion rate was high (97.5%) and missing data rates were low (< 1.5% for all items). The ordering of item means within scales generally were clustered as hypothesized and scaling assumptions were satisfied. Known groups analysis showed good discriminant validity between subgroups of healthy persons with differing health states. However, some areas of concern were revealed. Possible translation problems of the Physical Functioning (PF) items were indicated by the comparatively low ceiling effects. High ceiling and floor effects were seen in both role functioning scales, possibly due to the dichotomous format of their response choices. The Social Functioning scale had a low reliability of 0.55, which may be due to cultural differences in the concept of social functioning. The Vitality scale correlated better with the Mental Health scale than with itself, possibly because a healthy mental state is central to the concept of vitality in Thailand.

**Conclusion:**

The summated ratings method can be used for scoring the Thai SF-36. The instrument was found to be reliable and valid for use in a general non-clinical population. Version 2 of the SF-36 could improve ceiling and floor effects in the role functioning scales. Further work is warranted to refine items that measure the concepts of social functioning, vitality and mental health to improve the reliability and discriminant validity of these scales.

## Background

Since its translation to Thai in 2000[1], the SF-36 Health Survey had been used extensively for assessing health-related quality of life (QOL) in Thai patients with a range of health conditions. It was used to evaluate functional status in depressive patients [2], mental health problems following the 2004 tsunami[3], QOL in postmenopausal women with bladder problems[4] as well as in patients with allergic rhinoconjunctivitis [5], severe cardiac failure[6] and sleep apnea[7]. Given the increasing popularity of the Thai SF-36, it is important to be assured that the psychometric properties required for valid interpretation of the SF-36 scores have been retained in the translation process.

Reliability and construct validity of the Thai SF-36 had been tested in several studies. Internal consistency reliability was assessed in cardiac patients[1] and in patients with low back pain[8]. Recent studies of patients with knee osteoarthritis [9,10] and of patients with allergic rhinoconjunctivitis reported on reliability and concurrent validity of the instrument. The Thai SF-36 was also used as the concurrent measure to determine the construct validity of other disease-specific QOL instruments (endstage renal failure[11]; chronic liver failure[12]). These studies concluded that the Thai SF-36 was reliable and valid for assessing QOL in Thailand.

Although all of these studies used the summated ratings method[13] to score the Thai SF-36 scales, none had verified that the Thai translation satisfied the scaling assumptions required to validate use of summated ratings scores[13]. Other Asian translations of the SF-36, although generally successful, had reported problems which were revealed through psychometric tests. Discriminant validity, particularly between the concepts of mental health and vitality, was of some concern in a Chinese and a Japanese translation[14,15]. Watkins [16] noted minor problems with internal consistency in several of the scales in a Vietnamese translation. These problems were attributed to cultural differences in the definition or structure of health and refinement of the translations recommended.

The primary purpose of this paper was to perform, on the Thai SF-36, tests of data quality, scaling assumptions, reliability and validity according to the methods outlined by the IQOLA Project [13]. A secondary purpose was to examine the reliability and validity of the instrument when applied to a large non-clinical general population sample of men and women enrolled with the Sukhothai Thammathirat Open University (STOU).

## Methods

### Data collection

The study took place in July 2005 and involved distance-education students of the STOU from all areas in Thailand who were in Bangkok for pre-graduation orientation. The students were invited to complete a 4-page questionnaire comprising the Krittaphong translation of the Thai SF-36 (Version 1)[1] and a few questions on socio-demographic characteristics. The questionnaires were self-administered and students returned completed questionnaires to administrative personnel. This study was approved by the Ethics Committee of the Australian National University (protocol 2004344) and the Research and Development Institute of STOU (no 0522/10).

Of the 1388 students who returned the survey, 97.5% completed the questionnaire. The 43 incomplete questionnaires with entire pages left unanswered were not included in the following analyses.

About half of the respondents (744) had participated also in the baseline survey of an STOU-wide cohort study begun earlier in 2005. This survey had sought wide-ranging information on social demography, work, health service use, disease and injury, social factors, environment, food, physical activity, smoking and alcohol[17]. Selected health-related information from this survey was used to perform known-groups validity tests.

### Coding of items and scales

The SF-36 Health Survey is a generic questionnaire consisting of 36 items clustered to measure eight health concepts: Physical Functioning (PF), Role Limitations due to Physical Health (Role-Physical, RP), Bodily Pain (BP), General Health Perceptions (GH), Vitality (VT), Social Functioning (SF), Role Limitations due to Emotional Problems (Role-Emotional, RE) and Mental Health (MH). There is in addition a single-item measure of Health Transition (HT).

#### Item (raw) scores

Response choices for the items were on 2-, 3-, 5- or 6-point scales. Item scores ranged from 1 to 2, 3, 5 or 6 and were recoded so that all items scored in the same direction, with higher values indicating fewer limitations or better health states.

#### Scale scores

The SF-36 scales were scored using the method of summated ratings which assumes that items within a hypothesized scale can be summed without score standardization or item weighting [13]. Each scale was scored from 0 (worst possible health state) to 100 (best possible health state) by transforming and averaging the transformed scores[13]. The transformed score equaled 100× (observed item score – lowest possible item score)/(highest possible item score – lowest possible item score). A missing value was assigned to a scale when more than half of the items were missing. Where fewer items were missing, they were replaced by the respondent's own mean score for the remaining items on the scale.

### Analytic methods

#### Data quality

The number of completed items, the percent of missing data in every item and the frequency distribution of individual items were determined.

#### Ordering of levels of health

The ordering of item means within its scale was examined and compared with hypothesized orderings. Ware et a l[18] hypothesized that it was less likely for people to achieve higher than lower levels of a function or to endorse positive than negative health states. An item that measures a higher level of function should have a lower mean than one that measures a lower level of function. Items within a scale were put into clusters. Each cluster comprised items measuring similar levels of function. Items within the same cluster should have similar means and no ordering was hypothesized. If each translated item of the Thai SF-36 defined the same level of health as the original SF-36, the item means should cluster in the same order as hypothesized for the original SF-36.

#### Tests of scaling assumptions

Tests of scaling assumptions determine the appropriateness of including an item in a particular scale and the validity of using the summated ratings algorithm to construct scale scores. Four tests were conducted:

1. *Equal item variance*: Items measuring the same concept should have roughly equal standard deviations and should be around 1.0 (for 5-choice response scales) [13].

2. *Equality of item-scale correlations*: Items in each scale should contain approximately the same proportion of information about the concept being measured. This property was assessed by examining the correlation of an item with its hypothesized scale after correcting for overlap. Correction for overlap is necessary because ordinary correlations between an item and the scale of which it is a part are spuriously inflated. The method of Cureton [19] was used, wherein the item in question was replaced by a rationally equivalent item [19].

3. *Item internal consistency*: An item should measure what its scale is intended to measure (internal consistency). This property would be demonstrated by a scale if the item-scale correlations, corrected for overlap, of all items in the scale were 0.4 or greater.

4. *Item discriminant validity*: The correlation of each item with its hypothesized scale should be significantly higher than correlations of the same item with other scales. Item discriminant validity was supported, and the test considered a "definite success"[20], if item-scale correlations, corrected for overlap, were at least two standard errors above the correlations between that item and all the other scales. The standard error (SE) used was the SE for a correlation coefficient, which is approximately one divided by the square-root of the sample size. Seven item discriminant validity tests were conducted for each item.

After performing the item-level analyses above, summated rating scales were constructed and scale-level analyses were carried out. These included examination of scale-level properties, reliability and construct validity.

#### Statistical properties

The five scales which primarily measure disability (PF, RP, BP, SF, RE) should have the highest mean scale scores, while lower mean scores should be found for the three scales which extend measurement to the well-being range (GH, VT, MH). In order for a scale to include all important levels of the concept it measures, scale scores should have substantial variability and the full range of the measure should be used. The percentage of respondents with scores at the ceiling (score of 100) and floor (score of 0) were calculated for each scale. Ceiling and floor effects should be less than 20% to ensure that the scale is capturing the full range of potential responses in the population and that changes over time can be detected.

#### Reliability

Internal consistency reliability was estimated with the Cronbach α coefficient. It is a measure of the extent to which items within the same scale correlate with each other. It can be thought of as a correlation between a scale and itself. The α coefficient ranges from 0 to 1: values greater than 0.70 are generally considered acceptable for group comparisons, and 0.90 for person-level comparisons [13].

#### Construct validity

Construct validity was assessed by examining the correlations between the scales and by checking "known groups" validity[21]. Substantial correlation (Pearson's r > 0.40) was hypothesized between scales that were conceptually related (convergent validity). To evaluate how distinct each scale was from other scales (divergent validity), inter-scale correlations were compared with internal consistency reliability coefficients. Known groups validity was tested by comparing scale scores, adjusted for age and sex, across groups known to differ. SF-36 scores were hypothesized to be lower in persons with disabling health-related conditions; specifically depression/anxiety, arthritis, impaired vision not correctable by refraction and problems with eating, chewing or swallowing caused by teeth or dentures. These tests were performed on the sample of 744 participants using data from the cohort baseline survey.

## Results

Median age of the analysis sample was 31 years. The range spanned 21 to 78 years, with more than 85% under 40 years. Almost two-thirds (61.4%) were females.

### Data quality

The percent of missing item-level data was low – 32 of the 36 items showed less than 1% missing (Table 1). All of the response choices were used. The percent of respondents with computable scale scores was high: over 99% of respondents for seven scales, and 98.9% for the SF scale.

**Table 1:** Item percent missing, item means and standard deviations (SD)^a^

**Scale**	**SF-36 Item**		**% Missing**	**Mean**	**SD**
Physical Functioning (PF)					
	Vigorous activities	PF1	1.1	1.99	0.64
	Walking more than a kilometer	PF7	1.0	2.22	0.73
	Climbing several flights of stairs	PF4	1.0	2.51	0.63
	Bending, kneeling, stooping	PF6	1.0	2.56	0.59
	Lifting or carrying groceries	PF3	1.2	2.56	0.61
	Moderate activities	PF2	0.5	2.58	0.57
	Walking more than 100 m	PF8	0.7	2.65	0.58
	Climbing one flight of stairs	PF5	1.3	2.74	0.50
	Walking 100 m	PF9	1.3	2.76	0.50
	Bathing or dressing	PF10	0.5	2.90	0.33
Role-Physical (RP)					
	Accomplished less than would like	RP2	0.1	1.82	0.38
	Difficulty performing work/activities	RP4	0.2	1.76	0.43
	Cut down time spent on work	RP1	0.2	1.83	0.38
	Limited in kind of work/activities	RP3	0.2	1.88	0.33
Bodily Pain (BP)					
	Intensity of bodily pain	BP1	0.3	4.51	1.11
	Extent pain interfered with work	BP2	0.7	4.24	0.76
General Health (GH)					
	Rating of general health	GH1	0.0	3.07	0.78
	My health is excellent	GH5	0.7	3.58	1.09
	I seem as healthy as anyone I know	GH3	0.3	3.96	0.98
	I seem to get sick easier than others	GH2	0.7	3.76	1.12
	I expect my health to get worse	GH4	0.7	3.80	1.12
Vitality (VT)					
	Have a lot of energy	VT2	0.5	3.62	0.98
	Full of life	VT1	0.5	3.75	0.93
	Feel worn out	VT3	0.6	4.52	0.88
	Feel tired	VT4	0.2	4.55	0.91
Social Functioning (SF)					
	Extent health problems interfered	SF1	0.5	4.31	0.75
	Frequency health problems interfered	SF2	0.7	3.94	0.98
Role-Emotional (RE)					
	Accomplished less than would like	RE2	0.5	1.77	0.42
	Cut down time spent on work	RE1	0.4	1.78	0.42
	Work not done as carefully as usual	RE3	0.5	1.87	0.34
Mental Health (MH)					
	Felt calm and peaceful	MH3	0.5	3.30	0.93
	Been a happy person	MH5	0.6	4.11	0.98
	Been a very nervous person	MH1	0.2	4.35	0.85
	Felt down hearted and blue	MH4	0.5	4.85	0.92
	Felt down in the dumps	MH2	0.5	4.92	0.92
Health Transition (HT)					
	Change in health from one year ago	HT	0.2	2.88	0.84

^a^Items within a scale are ordered according to their relative expected means[20], with items having the highest expected mean at the top.

### Ordering of item means

The ordering of item means within each scale was consistent with hypothesized expectations along the health continuum (Table 1). Within the PF scale, the most difficult item (PF1: vigorous exercise) had the lowest mean and the easiest item (PF10: bathing and dressing) had the highest mean. Item means decreased across clusters of PF items as hypothesized; for example respondents reported more limitations (lower mean score) in climbing several stairs (PF4) than one flight of stairs (PF5).

Within the VT scale, items that measured energy or well-being (VT1 and VT2) had lower means than items measuring fatigue or disability (VT3 and VT4) as hypothesized. Within the MH scale, items measuring positive affect (MH3 and MH5) had lower means than items measuring negative affect (MH1, MH2 and MH4).

The two role functioning items that asked if the respondent "accomplished less" (RP2 and RE2) were hypothesized to have the lowest mean within its scale. This was observed for RE2 within the RE scale, but RP2 did not have the lowest mean in the RP scale. The only other item whose order was not as hypothesized was GH3 ("healthy as anyone I know").

The mean score for the Health Transition item was 2.88, indicating that respondents on average rated their health marginally worse than a year ago.

### Tests of scaling assumptions

Standard deviations of items within a scale were similar and close to 1.0 for BP, GH, VT, SF and MH (scales with 5- and 6-choice responses).

**Figure 1:**
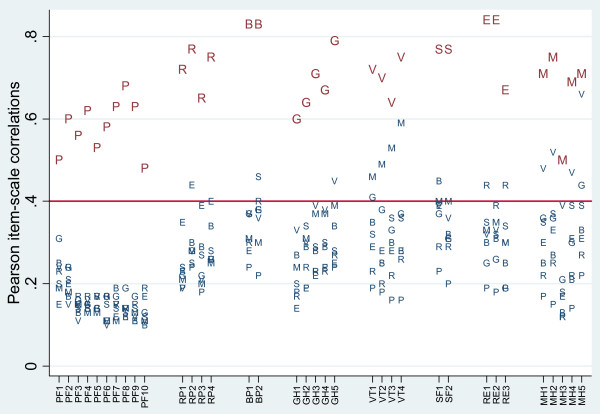
**Thai SF-36 item-scale correlations**. The horizontal axis shows the individual items; the vertical axis shows item-scale correlations. Correlations are labelled with letters to indicate the scale (P = PF, R = RE, B = BP, G = GH, V = VT, S = SF, E = RE, M = MH). Correlations are displayed in large font for hypothesized scales and in smaller font for non-hypothesized scales.

Figure 1 summarises the results visually for the other three scaling assumption tests. For all but two scales, correlations of items with their hypothesized scales were roughly equal. The item-scale correlations of all items were 0.08 units or less from at least one other item-scale correlation within its scale, except the item-scale correlations of RE3 and MH3 which were 0.17 and 0.19 units respectively from the next closest item-correlations in their scales. All item-scale correlations were greater than 0.40. The success rate for the item internal consistency test was 100% for all scales (Table 2). Looking at the distances between item correlations with their hypothesized scales and correlations of the same item with the non-hypothesized scales, the smallest distance was 0.11, between the MH5-MH correlation and the MH5-VT correlation (Figure 1), which was greater than two standard errors apart. This implied that all items achieved "definite scaling success" (Table 2).

**Table 2:** Tests of scaling assumptions

Scale	# items per scale, k	(a) Item internal consistency			(b) Item discriminant validity		
			
		Range^a^	Comparison^b^	Success rate (%)	Range^c^	Comparison^d^	Success rate (%)
PF	10	0.48 – 0.68	10/10	100	0.10 – 0.31	70/70	100
RP	4	0.65 – 0.77	4/4	100	0.18 – 0.44	28/28	100
BP	2	0.83	2/2	100	0.22 – 0.46	14/14	100
GH	5	0.60 – 0.79	5/5	100	0.14 – 0.45	35/35	100
VT	4	0.64 – 0.75	4/4	100	0.16 – 0.59	28/28	100
SF	2	0.77	2/2	100	0.20 – 0.40	14/14	100
RE	3	0.67 – 0.84	3/3	100	0.18 – 0.44	21/21	100
MH	5	0.50 – 0.75	5/5	100	0.12 – 0.66	35/35	100

^a ^Correlations between items and hypothesized scale, corrected for overlap

^b ^Number of items out of k with correlation ≥ 0.40

^c ^Correlations between items and other scales

^d ^Number of items out of 7 × k where difference between the correlation of the item with its own scale and correlation with the other scales ≥ 2SE (= 0.0576)

### Scale properties

As hypothesized, the scales measuring both positive and negative aspects of well-being (GH, VT and MH) produced lower mean scores than the scales measuring disability (PF, RP, BP, SF and RE) (Table 3).

**Table 3:** Descriptive statistics for the eight scales

Scale	Range	Median	Mean	SD	Skewness	%Floor	%Ceiling
PF	0 – 100	80	77.3	17.4	-1.04	0.3	8.7
RP	0 – 100	100	82.2	28.6	-1.54	4.5	64.9
BP	10 – 100	77.5	75.6	18.4	-0.46	0	20.6
GH	0 – 100	65	65.1	18.1	-0.56	0.2	0.6
VT	0 – 100	60	62.2	13.3	-0.30	0.1	0.3
SF	0 – 100	75	78.2	18.2	-0.58	0.1	26.0
RE	0 – 100	100	80.4	31.9	-1.41	7.4	67.4
MH	8 – 100	68	66.1	12.9	-0.48	0	0.4

The distributions of scores showed good spread, with the full 0–100 range observed in six of the eight scales (Table 3). As expected for a sample primarily composed of healthy respondents, response distributions tended to be skewed in the direction of positive health (relatively high median and negative skewness). The relatively low mean of 77.3 for PF was surprising, given the relative youth and health of the sample, as was its low ceiling effect of 8.7%. The percentage of respondents scoring the lowest scale level (floor effect) was minimal. Floor effects were observed in less than 1% of the sample for all but the two role functioning scales (RP and RE). The dichotmous response format of the RP and RE scales also resulted in these scales exhibiting substantial ceiling effects (> 60%). The scales which measure both disability and well-being (GH, VT, MH) showed minimal floor and ceiling effects.

### Reliability

Internal consistency reliability estimates of six of the eight scales exceeded the 0.70 level recommended for group comparisons, though none met the criterion for person-level comparisons (Table 4). The reliability estimate for the SF scale was low (0.55); that for the VT scale (0.68) was only marginally below the 0.70 criterion.

**Table 4:** Inter-scale correlations and internal consistency reliability (Cronbach α coefficients, on the diagonal)

	PF	RP	BP	GH	VT	SF	RE	MH
PF	*0.80*							
RP	0.29	*0.75*						
BP	0.23	0.38	*0.74*					
GH	0.29	0.32	0.41	*0.75*				
VT	0.21	0.30	0.39	0.51	*0.68*			
SF	0.24	0.34	0.45	0.39	0.44	*0.55*		
RE	0.21	0.51	0.35	0.28	0.37	0.39	*0.73*	
MH	0.21	0.29	0.33	0.47	0.71	0.47	0.39	*0.74*

### Validity

Higher coefficients were found between scales which represent similar constructs (eg MH and VT) than those with competing constructs (eg PF and RE). Comparisons of inter-scale correlations revealed that the scale constructs were generally distinct: most of the inter-scale correlation coefficients were low to medium (0.21 to 0.51). The exception was an inter-scale correlation of 0.71 between the VT and the MH scales.

All SF-36 scores were higher in persons without the disabling health condition than in persons with the condition (Table 5). In the comparison of depression or anxiety, scales which showed statistical significance tended to be those relating to mental health, while in the comparison of arthritis, scales relating to physical health showed statistically significance.

**Table 5:** Comparison of scale scores between persons with and without selected health conditions^a^

	**%**	**PF**	**RP**	**BP**	**GH**	**VT**	**SF**	**RE**	**MH**
**Depression^1^**									
No	96	77.5 (17.4)	82.2 (28.5) #	74.9 (18.2)	65.2 (17.4) $	61.8 (13.5) #	78.3 (18.5) #	80.2 (31.8) $	65.5 (13.0) $
Yes	4	71.7 (14.8)	65.1 (37.5)	70.0 (19.1)	48.6 (22.0)	53.7 (13.2)	66.7 (17.8)	52.6 (41.6)	56.4 (12.7)
**Arthritis^1^**									
No	95	77.7 (16.9) *	82.9 (28.4) $	75.3 (18.1) #	65.0 (17.8)	61.6 (13.6)	77.9 (18.6)	79.3 (32.5)	65.3 (12.9)
Yes	5	71.1 (22.8)	61.5 (31.9)	65.8 (18.1)	58.6 (17.4)	59.1 (12.7)	77.3 (18.8)	78.7 (33.0)	61.9 (15.9)
**Impaired vision not correctable by glasses/contact lens^2^**									
No	90	78.0 (16.9)	84.1 (27.5) *	76.0 (18.3)	66.8 (17.4) #	62.7 (13.4) #	79.4 (18.2) *	81.8 (31.0)	66.5 (13.1) #
Yes	10	75.1 (18.2)	76.5 (33.3)	74.0 (16.9)	60.4 (17.2)	58.0 (14.4)	74.7 (19.9)	74.3 (35.9)	62.0 (13.2)
**Problems caused by teeth or dentures^3^**									
No	71	79.0 (16.8) #	84.2 (27.4)	77.3 (18.1) $	67.8 (17.7) $	62.9 (13.6) *	79.5 (18.7)	82.6 (31.1) *	66.9 (13.2) #
Yes	29	75.4 (16.6)	81.0 (30.3)	71.6 (18.2)	62.3 (17.6)	60.4 (13.6)	77.1 (18.4)	77.0 (31.6)	63.7 (13.2)

^a^Cells show mean (standard deviation). Symbols beside figures indicate statistical significance of the comparison: * = p < 0.01; # = p < 0.001; $ = p < 0.0001

^1^Based on self-report to the question "Ever been told by a doctor that you have this condition"

^2^Answer to the question "Do you currently have any sight problems not correctable by glasses/contact lenses (eg cataract)"

^3^In response to the question "Do your teeth currently cause you...", ticking "yes" to any of the conditions "discomfort speaking", "discomfort swallowing", "discomfort chewing" or "pain".

## Discussion

This paper demonstrated that psychometric properties of the Thai SF-36 were satisfactory according to the criteria set by the IQOLA project protocol. In particular, the Thai SF-36 can be scored using the summated ratings method. The results have added to existing evidence that the concepts embodied in the SF-36 are applicable to the Thai population.

Overall data quality was satisfactory. Questionnaire completion rate (97.5%) was high and compared favourably with rates ranging from 88% to 99% reported for self-administered surveys of the SF-36 in other countries [21]. Of the 43 respondents who missed pages, most had omitted questions on the reverse side of the page and the remainder answered only the first few pages. Missing data rates (< 1.5% for all items) were low. Use of all of the response choices for all 36 items suggested that translations of all response choices and the associated items were understood.

The ordering of item means within scales generally were clustered as hypothesized, with two exceptions involving the "role-physical accomplished less" (RP2) and "healthy as anyone I know" (GH3) items. The deviation of RP2 was small, only 0.06, so not surprising given the coarse structure of the dichotomous response choices. Similar deviations of GH3 observed in other studies[20,22] were attributed to the difference in construction of GH3, which measures health relative to other people, and the construction of GH1 and GH5, which measure absolute health.

Results of the scaling assumption tests basically supported the hypothesized scale structure of the SF-36 in Thailand and use of the summated ratings algorithm. The only scaling assumption not fully satisfied was the lack of equality in the item-scale correlations of RE3 and the other RE items and of MH3 and the other MH items. Other studies had found similar discrepancies; e.g. [16,22]. These discrepancies were not considered significant problems as Ware & Gandek[13]'s view was that: "when all items contribute fully to the total score, this standard [equality of item-scale correlations] can be considered fully satisfied even if item-scale correlations vary".

A few areas warrant further examination. Unlike most other general population samples (for example, [15,22-24]) the mean PF scale score in this study was higher than the mean scale scores of RP and BP. The ceiling effect of the PF scale (8.7%) was also lower than in other general population samples which were typically greater than 20%[20]. These differences suggested the possibility of translation problems in the PF scale.

The high ceiling effects in the two role functioning scales (RP 79%; RE 77.3%) could be explained, at least partly, by the dichotomous format of the items comprising these scales. Similar results had been observed in many other studies; for example in Gandek's comparison of 11 countries [21], ceiling effects ranged from 63.3% to 82.9% for RP and from 69.0% to 82.8% for RE. The limitations of these dichotomous items could be minimized by extending the response choices, such as the 5-point Likert response in Version 2 of the SF-36.

Except for the SF scale, internal consistency reliability was generally acceptable for group-level comparisons. Low reliability of the SF scale had been observed in elsewhere including several Asian studies. Chinese translations reported reliabilities of 0.39, 0.54, 0.57 and 0.65[15,22,23,25]; 0.67 was found in a Vietnamese translation [16] and 0.68 in a Japanese translation[20]. In Asian cultures translation of these items had been reported to be difficult because of cultural differences in the concept of social functioning. Wagner [26] reported on the high difficulty ratings in translation of the SF items in a cross-cultural comparison of 10 countries.

The correlations between scales generally were less than the within-scale correlations (reliability coefficient). This was indication that the Thai SF-36 scales generally could discriminate between the different concepts being measured, excepting the concepts of vitality and mental health. Although both the VT and MH items individually had higher correlations with their hypothesized scales than with other scales, the VT scale was found to correlate higher with the MH scale than with itself. Several other studies had also reported moderately high correlations (over 0.60) between these scales [15,24,25]. In a cross-country comparison of primarily Western countries, Gandek et al[20] attributed the substantial correlations observed to a "method effect" due to the different constructions of some of the items in the two scales. In the Asian studies[15,25], however, the high correlations between the VT and MH scales were attributed to cultural differences where happiness and a healthy mental state were central to the concept of vitality. When evaluating a Chinese translation, Chang et al[27] suggested that the vitality and mental health items could be more meaningfully reorganized along the dimensions of well-being and distress. Watkins et al [16], in developing a Vietnamese translation, had modified the conceptual definition of the MH and VT scales to produce culturally more appropriate scales with clearer delineation between these concepts. For Thai people, who like Vietnamese and mainland Chinese are predominantly Buddhists, a healthy mental state is fundamental to vitality. Further work to refine the items measuring these concepts is warranted.

Previous studies had reported that the Thai SF-36 could discriminate between different levels of ill health in clinically ill subjects[1,2,4,5,9]. Known groups analysis in this study indicated that the Thai SF-36 also discriminated well between generally healthy persons who differed in health states. Persons who had depression, arthritis, impaired vision or difficulty eating scored significantly lower on several of the SF-36 scales.

This study had two main limitations. First, generalisability of the results to all of Thailand is limited as this study was conducted on a convenience sample of STOU students and would not be representative of the general population in Thailand. Second, data quality and acceptability of the instrument could have been over-estimated as assessments could be performed only on the questionnaires which were returned.

## Conclusion

The present study has provided valuable additional evidence that supports use of the Thai SF-36. The results have filled a gap by confirming that the summated ratings method can be used to score the Thai SF-36. Reliability and validity were established for use of the instrument in the general population. Problems revealed through the psychometric tests indicated that there may be some translation problems with the Physical Functioning scale, that ceiling and floor effects could be reduced with use of Version 2 of the SF-36, and that refinement of items in the Social Functioning, Vitality and Mental Health scales could improve reliability and discriminant validity of these scales.

## Abbreviations

BP: Bodily Pain; GH: General Health; IQOLA: International Quality of Life Assessment; MH: Mental Health; PF: Physical Functioning; QOL: Quality of Life; RE: Role-Emotional; RP: Role-Physical; SF-36: Short Form 36; SF: Social Functioning; STOU: Sukhothai Thammathirat Open University; VT: Vitality.

## Competing interests

The authors declare they have no competing interests.

## Authors' contributions

LL, SS and AS jointly conceived the study. LL performed the statistical analysis and drafted the manuscript. SS designed, managed and coordinated the study. AS participated in the study conduct and manuscript preparation. All authors read and approved the final manuscript.
